# Eagle syndrome masquerading as a chicken bone

**DOI:** 10.1186/s12245-020-0262-7

**Published:** 2020-01-13

**Authors:** Jason E. Cohn, Sammy Othman, Karima Sajadi-Ernazarova

**Affiliations:** 1 Department of Otolaryngology-Head and Neck Surgery, Philadelphia College of Osteopathic Medicine, 4190 City Line Avenue, Philadelphia, PA 19131 USA; 2 Department of Emergency Medicine, Drexel University College of Medicine, Philadelphia, PA USA; 3 Drexel University College of Medicine, Philadelphia, PA USA

**Keywords:** Eagle syndrome, Elongated stylohyoid ligament, Calcified stylohyoid ligament, Chicken bone, Plain radiograph

## Abstract

This is a brief report of a 17-year-old male who presented to the emergency department with odynophagia and a foreign body sensation in the throat after choking on a chicken wing. A soft tissue neck radiograph was performed which revealed a 4.6-cm linear object in the vallecula read by the radiology department as a chicken bone. The otolaryngology team was consulted and performed a nasopharyngeal laryngoscopy which did not reveal a foreign body in the upper aerodigestive tract. On physical examination, the right tonsillar fossa was tender to palpation. Upon further review of the radiograph, the right stylohyoid ligament was noted to be elongated and calcified. Thereafter, the diagnosis of Eagle syndrome was made. This case provides an important teaching point for providers by pointing out a syndrome that can mimic other disease processes. In addition, it emphasizes the importance of providers reviewing their own films.

## Case presentation

**Fig. 1:**
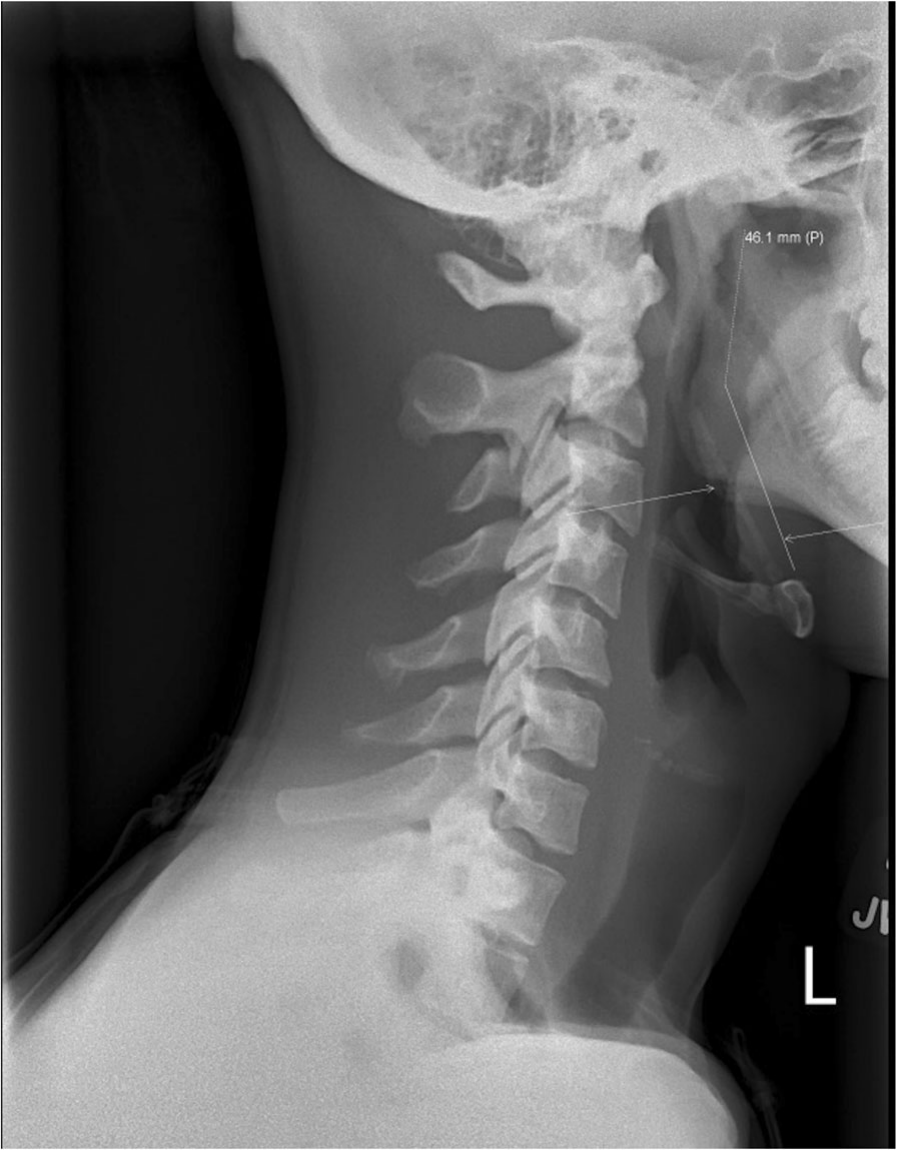
Elongated and calcified stylohyoid ligament on lateral neck radiograph (arrow) measuring 4.6 cm

A 17-year-old male presented to the emergency department with odynophagia and a foreign body sensation in the throat after choking on a chicken wing. A soft tissue neck radiograph was performed which revealed a 4.6-cm linear object in the vallecula read by the radiology department as a chicken bone. The otolaryngology team was consulted and performed a nasopharyngeal laryngoscopy which was unremarkable. On physical examination, the right tonsillar fossa was tender to palpation. Upon further review of the radiograph, the right stylohyoid ligament was noted to be elongated and calcified (Fig. 1).

## Diagnosis

### Eagle syndrome

Although this patient presented after choking on a chicken bone, there was no foreign body present in the aerodigestive tract. The key symptom of foreign body sensation can be a classic presentation of Eagle syndrome.

Eagle syndrome is a symptomatic elongation with either overgrowth of the styloid process itself or ossification of the stylohyoid ligament complex [1]. Symptoms can include a constant dull pharyngeal pain, focused in the ipsilateral tonsillar fossa, that can be referred to the ear and aggravated by rotation of the head as well as the sensation of a foreign body in the pharynx, dysphagia, odynophagia, headache, and tinnitus [1, 2].

The diagnosis can be made on plain radiograph, but CT is the most accurate [1–3]. The normal length of the adult styloid is approximately 2.5 cm while greater than 3 cm is considered elongated [1]. Treatment options include medical therapies (typically analgesics) and transoral or external surgeries (i.e., styloidectomy) [1, 2].

## Data Availability

Not applicable

## References

[1] Bokhari MR, Mohseni M (2018). Eagle syndrome. StatPearls [Internet].

[2] Badhey A, Jategaonkar A, Anglin Kovacs AJ, et al. Eagle syndrome: a comprehensive review. Clin Neurol Neurosurg. 2017 Aug;159:34-38. doi: 10.1016/j.clineuro.2017.04.021. Epub 2017 May 6. 10.1016/j.clineuro.2017.04.021 28527976

[3] Murtagh RD, Caracciolo JT, Fernandez G (2001). CT findings associated with Eagle syndrome. AJNR Am J Neuroradiol..

